# A risk stratification model to predict chemotherapy benefit in medullary carcinoma of the breast: a population-based SEER database

**DOI:** 10.1038/s41598-023-37915-2

**Published:** 2023-07-03

**Authors:** Heyan Chen, Shengyu Pu, Lizhao Wang, Huimin Zhang, Yu Yan, Jianjun He, Jian Zhang

**Affiliations:** Department of Breast Surgery, The First Affiliated Hospital of Xi’an Jiaotong University, 277 West Yanta Road, Xi’an, 710061 Shaanxi China

**Keywords:** Cancer, Oncology

## Abstract

Whether patients with medullary breast carcinoma (MBC) receive chemotherapy is controversial. Therefore, the aim of our study was to screen out patients with MBC who benefit from chemotherapy. We enrolled 618 consecutive patients with MBC from The Surveillance, Epidemiology, and End Results (SEER) database (2010–2018). Cox regression analysis was used to identify independent prognostic factors. Next, a nomogram was constructed and evaluated using calibration plots and the area under the curve (AUC) of receiver operating characteristic (ROC) curves. Kaplan‒Meier curves were used to evaluate the overall survival (OS) benefit of chemotherapy in different risk groups. A total of 618 MBC patients were involved in our study, and an 8:2 ratio was used to randomly split them into a training cohort (n = 545) and a validation cohort (n = 136). Next, a nomogram predicting 3- and 5-year OS rates was constructed based on the five independent factors (age at diagnosis, T stage, N status, subtype and radiation). The nomogram AUCs for 3- and 5-year OS (training set: 0.793 and 0.797; validation set: 0.781 and 0.823) and calibration plots exhibited good discriminative and predictive ability. Additionally, a novel risk classification system for MBC patients demonstrated that we do not have enough evidence to support the benefit effect of chemotherapy for the high-risk group as the result is not statistically significant (total population: *p* = 0.180; training set: *p* = 0.340) but could improve OS in the low-risk group (total population: *p* = 0.001; training set: *p* = 0.001). Our results suggested that chemotherapy should be selected more carefully for high-risk groups based on a combination of factors and that the possibility of exemption from chemotherapy should be confirmed by more clinical trials in the future.

## Introduction

Breast cancer is a highly heterogeneous tumor that displays inter- and intratumoral heterogeneity^1,2^. Medullary breast carcinoma (MBC) is a rare and distinct pathological subtype of breast carcinoma and is characterized by a syncytial cell growth pattern, lack of duct formation and lymphocytic cell infiltration, and rare tumor necrosis (< 25%)^3^. Based on the above characteristics, MBC is split into typical and atypical MBC, with the latter having a worse prognosis than the former^3^. MBC accounts for 1–7% of all breast cancers^4^, with a mean age ranging from 45 to 54 years^5^, and most of them (95%) belong to basal-like subtypes based on gene expression analysis^6^.

Although MBC has aggressive biological features and a basal-like phenotype, it has a more favorable prognosis than other common infiltrating ductal carcinomas^7,8^. The 10-year overall survival rate for MBC patients was 74%, whereas that of patients with negative lymph nodes was over 90%^9^. The high frequency of apoptosis and the presence of lymph node metastasis in less than 10% of MBC patients may account for the favorable prognosis of MBC patients^10,11^. MBC can be treated with breast-conserving surgery, modified or radical mastectomy followed by radiotherapy or chemotherapy, depending on stage^12^. However, previous studies have confirmed that general risk factors, which are of major prognostic importance in invasive ductal carcinoma (IDC), had little prognostic impact in MBC^8^, and the prognosis of MBC patients with the TNBC subtype is better than that of IDC patients with the triple-negative breast cancer (TNBC) subtype^6^. Additionally, some studies have reported that some MBC patients with the TNBC subtype cannot obtain a survival benefit from adjuvant chemotherapy^13–17^, considering that chemotherapy can cause strong side effects. Therefore, the question of whether adjuvant chemotherapy is necessary or beneficial for some MBC patients with the TNBC subtype was raised^16^. At present, the answer to this question is not provided by the relevant literature.

Therefore, the aim of our study was to screen out patients with MBC who benefit from chemotherapy. We collected MBC patients from The Surveillance, Epidemiology, and End Results (SEER) database and constructed a risk-stratified prediction model using a nomogram according to the clinicopathological features of patients with MBC. To provide a reference for clinicians to make clinical decisions and avoid overtreatment.

## Results

### Patient characteristics

**Figure 1:**
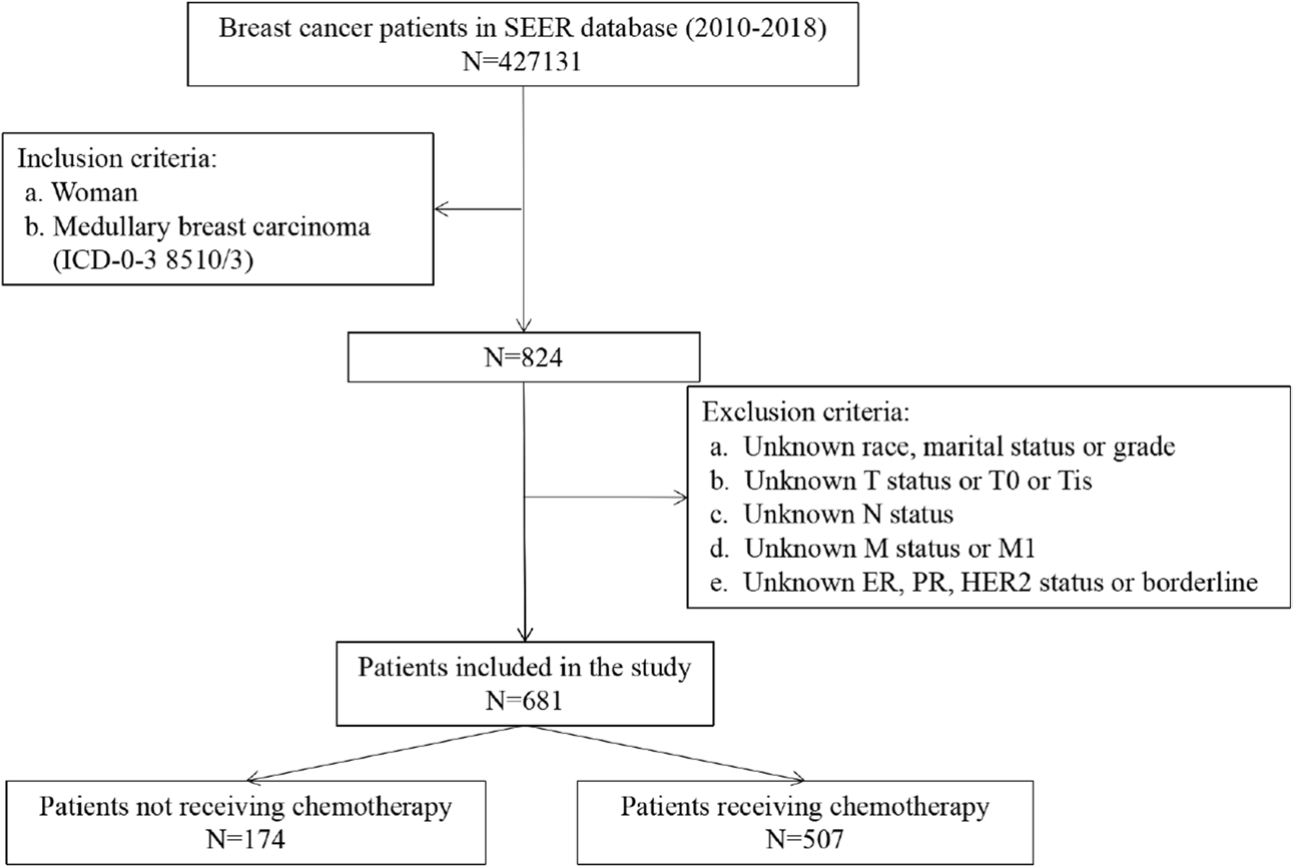
The flowchart of the population enrolled in our study. *ER* estrogen receptor, *PR* progesterone receptor, *HER2* human epidermal growth factor receptor.

A total of 681 eligible patients with MBC from the SEER database (2010–2018) were enrolled, including 174 MBC patients who were not undergoing chemotherapy (median follow-up time: 51.5 months) and 507 MBC patients who received chemotherapy (median follow-up time: 58 months). A screening flow diagram of our study population is presented in Fig. 1. The comparison of demographic and clinicopathological characteristics between the groups is shown in Table 1, which shows that compared with the nonchemotherapy group, patients in the chemotherapy group were younger, married, had a larger tumor size, had a higher lymph node metastasis rate and were more inclined to receive radiotherapy. However, there were no differences in the distribution of race, tumor grade, hormone receptor status, human epidermal growth factor receptor 2 (HER2) status, or molecular subtypes between the two groups.

**Table 1:** Clinicopathological characteristics of the study populations.

Characteristics	Before PSM			After PSM		
	No-chemotherapy	Yes-chemotherapy	*p* value	No-chemotherapy	Yes-chemotherapy	*p* value
No. patients	n = 174	n = 507		n = 174	n = 174	
Age at diagnosis (%)			< 0.001			0.845
< 40	14 (8.0)	92 (18.1)		14 (8.0)	14 (8.0)	
40–65	99 (56.9)	344 (67.9)		61 (35.1)	56 (32.2)	
> 65	61 (35.1)	71 (14.0)		99 (56.9)	104 (59.8)	
Race (%)			0.408			0.602
Black	46 (26.4)	128 (25.2)		46 (26.4)	54 (31.0)	
White	109 (62.6)	339 (66.9)		109 (62.6)	104 (59.8)	
Other	19 (10.9)	40 (7.9)		19 (10.9)	16 (9.2)	
Marital status (%)			0.003			0.913
Married	73 (42.0)	280 (55.2)		73 (42.0)	71 (40.8)	
Single	101 (58.0)	227 (44.8)		101 (58.0)	103 (59.2)	
Grade (%)			0.264			0.838
I–II	14 (8.0)	27 (5.3)		14 (8.0)	12 (6.9)	
III–IV	160 (92.0)	480 (94.7)		160 (92.0)	162 (93.1)	
T stage (%)			0.014			0.949
T1	90 (51.7)	201 (39.6)		90 (51.7)	92 (52.9)	
T2	77 (44.3)	269 (53.1)		77 (44.3)	76 (43.7)	
T3–T4	7 (4.0)	37 (7.3)		7 (4.0)	6 (3.4)	
N stage (%)			0.001			0.874
N0	152 (87.4)	380 (75.0)		152 (87.4)	150 (86.2)	
N1–N3	22 (12.6)	127 (25.0)		22 (12.6)	24 (13.8)	
ER status (%)			0.41			0.576
Negative	109 (62.6)	337 (66.5)		109 (62.6)	115 (66.1)	
Positive	65 (37.4)	170 (33.5)		65 (37.4)	59 (33.9)	
PR status (%)			0.491			0.409
Negative	138 (79.3)	416 (82.1)		138 (79.3)	145 (83.3)	
Positive	36 (20.7)	91 (17.9)		36 (20.7)	29 (16.7)	
HER2 status (%)			0.908			0.245
Negative	156 (89.7)	451 (89.0)		156 (89.7)	163 (93.7)	
Positive	18 (10.3)	56 (11.0)		18 (10.3)	11 (6.3)	
Subtype (%)			0.485			0.447
TNBC	90 (51.7)	288 (56.8)		90 (51.7)	102 (58.6)	
HER2-enriched	12 (6.9)	32 (6.3)		12 (6.9)	7 (4.0)	
Luminal A	66 (37.9)	163 (32.1)		66 (37.9)	61 (35.1)	
Luminal B	6 (3.4)	24 (4.7)		6 (3.4)	4 (2.3)	
Surgery (%)			0.568			1
No/unknown	5 (2.9)	9 (1.8)		5 (2.9)	4 (2.3)	
Yes	169 (97.1)	498 (98.2)		169 (97.1)	170 (97.7)	
Radiation (%)			< 0.001			1
No	114 (65.5)	224 (44.2)		114 (65.5)	115 (66.1)	
Yes	60 (34.5)	283 (55.8)		60 (34.5)	59 (33.9)	

*ER* estrogen receptor, *PR* progesterone receptor, *HER2* human epidermal growth factor receptor 2, *TNBC* triple-negative breast cancer, *PSM* propensity score matching.

**Figure 2:**
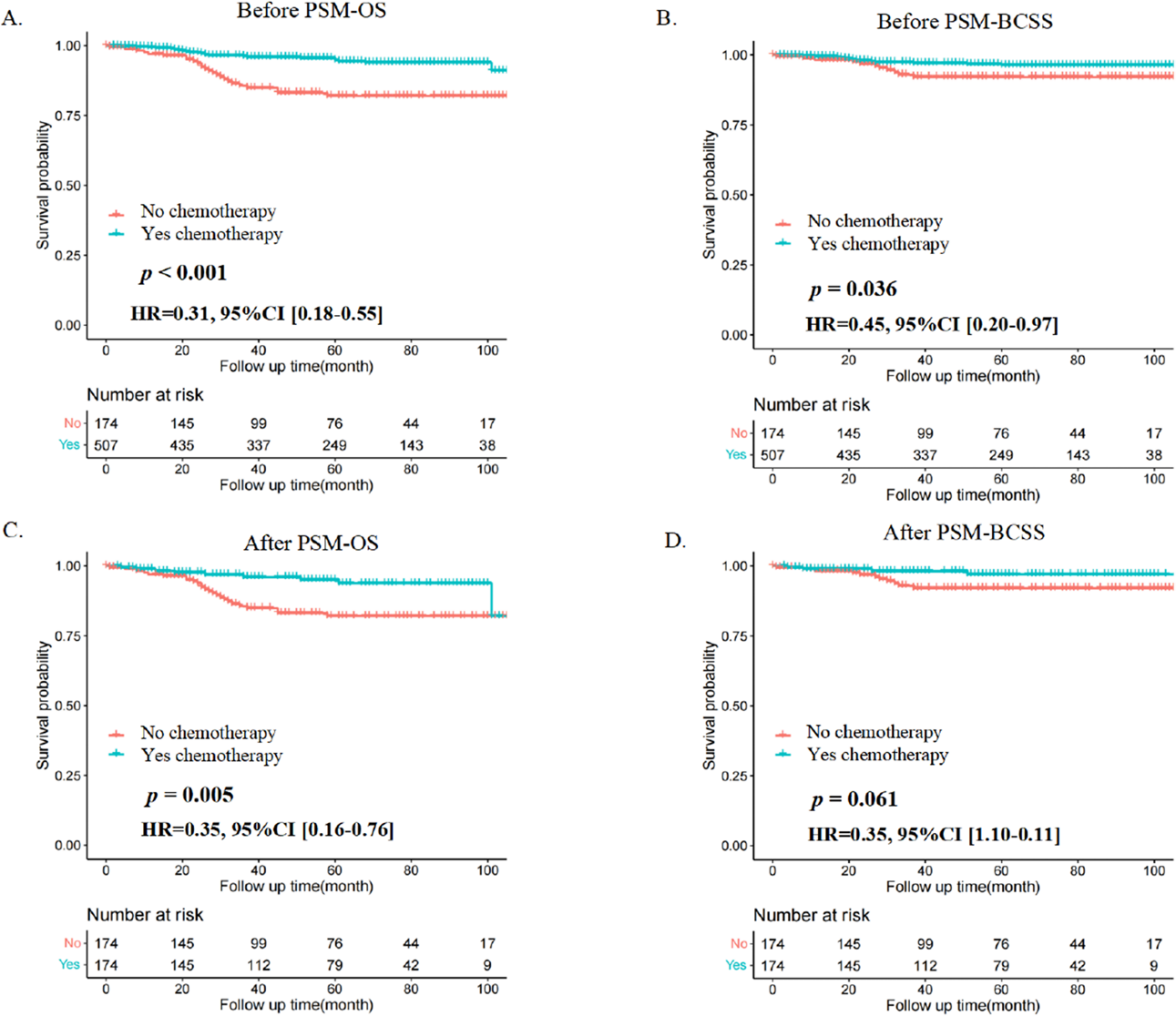
Kaplan‒Meier curves of OS and BCSS of the chemotherapy and non-chemotherapy groups in the total population. (**A**,**B**) Before PSM. (**C**,**D**) After PSM.

Additionally, as presented in Fig. 2A,B, patients with MBC who were not undergoing chemotherapy had unfavorable overall survival (OS) (*p* < 0.001) and breast cancer-specific survival (BCSS) (*p* = 0.036) compared to patients who received chemotherapy. Considering the impact of the difference in baseline distribution between the two groups, we conducted propensity score matching (PSM) analysis at a ratio of 1:1, and the difference disappeared after PSM (Table 1). At this point, we observed that after PSM, patients in the chemotherapy group retained better OS (*p* = 0.005) than those in the nonchemotherapy group (Fig. 2C), whereas the difference in BCSS (*p* = 0.061) between the two groups was not statistically significant (Fig. 2D).

### Independent prognostic factors

We further randomly split the total population into a training set (n = 545) and a validation set (n = 136) at a ratio of 8:2. As shown in Table 2, there was no variation in the demographic and clinicopathological traits were distributed between the two groups. Next, univariate and multivariate Cox analysis were developed subsequently in the training set (Table 3), which showed that age at dignosis > 65 years old (< 40 years old as a reference; > 65 years old: HR = 7.03, 95% CI (2.36–21.34), *p* = 0.001), T stage with T3–T4 (T1 as a reference; T3–T4: HR = 5.49, 95% CI (1.96–15.4), *p* = 0.001), and N stage with N1–N3 (N0 as a reference; N1–N3: HR = 2.39, 95% CI (1.19–4.8), *p* = 0.014) were independent unfavorable traits for OS of patients with MBC, whereas patients with Luminal A (TNBC as a reference; Luminal A: HR = 0.34, 95% CI (0.15–0.79), *p* = 0.012), and receiving radiotherapy (no radiation as a reference; radiation: HR = 0.34, 95% CI (0.15–0.79), *p* = 0.012) had better OS compared to those of TNBC and not receiving radiotherapy, respectively.

**Table 2:** Comparison of clinicopathological features between the training set and the validation set.

Characteristics	Training set	Validation set	p value
No. patients	n = 545	n = 136	
Age at diagnosis (%)			0.537
< 40	87 (16.0)	19 (14.0)	
40–65	349 (64.0)	94 (69.1)	
> 65	109 (20.0)	23 (16.9)	
Race (%)			0.493
Black	134 (24.6)	40 (29.4)	
White	364 (66.8)	84 (61.8)	
Other	47 (8.6)	12 (8.8)	
Marital status (%)			0.338
Married	288 (52.8)	65 (47.8)	
Single	257 (47.2)	71 (52.2)	
Grade (%)			1
I–II	33 (6.1)	8 (5.9)	
III–IV	512 (93.9)	128 (94.1)	
T stage (%)			0.391
T1	238 (43.7)	53 (39.0)	
T2	270 (49.5)	76 (55.9)	
T3–T4	37 (6.8)	7 (5.1)	
N stage (%)			0.771
N0	424 (77.8)	108 (79.4)	
N1–N3	121 (22.2)	28 (20.6)	
ER status (%)			0.134
Negative	349 (64.0)	97 (71.3)	
Positive	196 (36.0)	39 (28.7)	
PR status (%)			0.481
Negative	440 (80.7)	114 (83.8)	
Positive	105 (19.3)	22 (16.2)	
HER2 status (%)			0.694
Negative	484 (88.8)	123 (90.4)	
Positive	61 (11.2)	13 (9.6)	
Subtype (%)			0.156
HR−/HER2−	292 (53.6)	86 (63.2)	
HR−/HER2+	38 (7.0)	6 (4.4)	
HR+/HER2−	192 (35.2)	37 (27.2)	
HR+/HER2+	23 (4.2)	7 (5.1)	
Surgery (%)			1
No/unknown	11 (2.0)	3 (2.2)	
Yes	534 (98.0)	133 (97.8)	
Radiation (%)			0.443
No	266 (48.8)	72 (52.9)	
Yes	279 (51.2)	64 (47.1)	
Chemotherapy (%)			0.784
No/unknown	141 (25.9)	33 (24.3)	
Yes	404 (74.1)	103 (75.7)	

*ER* estrogen receptor, *PR* progesterone receptor, *HER2* human epidermal growth factor receptor 2, *TNBC* triple-negative breast cancer.

**Table 3:** Univariate and multivariate Cox analyses of OS in the training set.

Characteristics	Univariate analysis			Multivariate analysis		
	HR	95% CI	p value	HR	95% CI	p value
Age at diagnosis						
< 40	1			1		
40–65	1.03	0.34–3.09	0.956	1.17	0.39–3.54	0.778
> 65	4.45	1.52–13.02	0.006	7.03	2.36–21.34	0.001
Race						
Black	1			–		
White	0.63	0.32–1.24	0.180	–	–	–
Other	0.64	0.18–2.25	0.485	–	–	–
Marital status						
Married	1			1		
Single	1.94	1.03–3.65	0.041	1.48	0.78–2.81	0.228
Grade						
I–II	1			–		
III–IV	0.51	0.18–1.43	0.198	–	–	–
T stage				–	–	–
T1	1			1		
T2	1.70	0.82–3.50	0.152	1.68	0.8–3.53	0.174
T3–T4	5.07	1.96–13.08	0.001	5.49	1.96–15.4	0.001
N stage						
N0	1			1		
N1–N3	2.09	1.10–3.97	0.024	2.39	1.19–4.8	0.014
ER status						
Negative	1			–		
Positive	0.46	0.21–1.00	0.050	–	–	–
PR status				–	–	–
Negative	1			–		
Positive	0.32	0.10–1.05	0.060	–	–	–
HER2 status						
Negative	1			–		
Positive	1.10	0.43–2.82	0.837	–	–	–
Subtype						
TNBC	1			1		
HER2-enriched	1.04	0.37–2.98	0.935	0.61	0.21–1.8	0.375
Luminal A	0.39	0.17–0.89	0.026	0.34	0.15–0.79	0.012
Luminal B	0.47	0.06–3.47	0.461	0.54	0.07–4.08	0.551
Surgery						
No/unknown	1			–		
Yes	0.30	0.07–1.26	0.101	–	–	–
Radiation						
No	1			1		
Yes	0.36	0.18–0.72	0.004	0.28	0.14–0.57	0.001

*ER* estrogen receptor, *PR* progesterone receptor, *HER2* human epidermal growth factor receptor 2, *TNBC* triple-negative breast cancer.

### Nomogram development and validation

**Figure 3:**
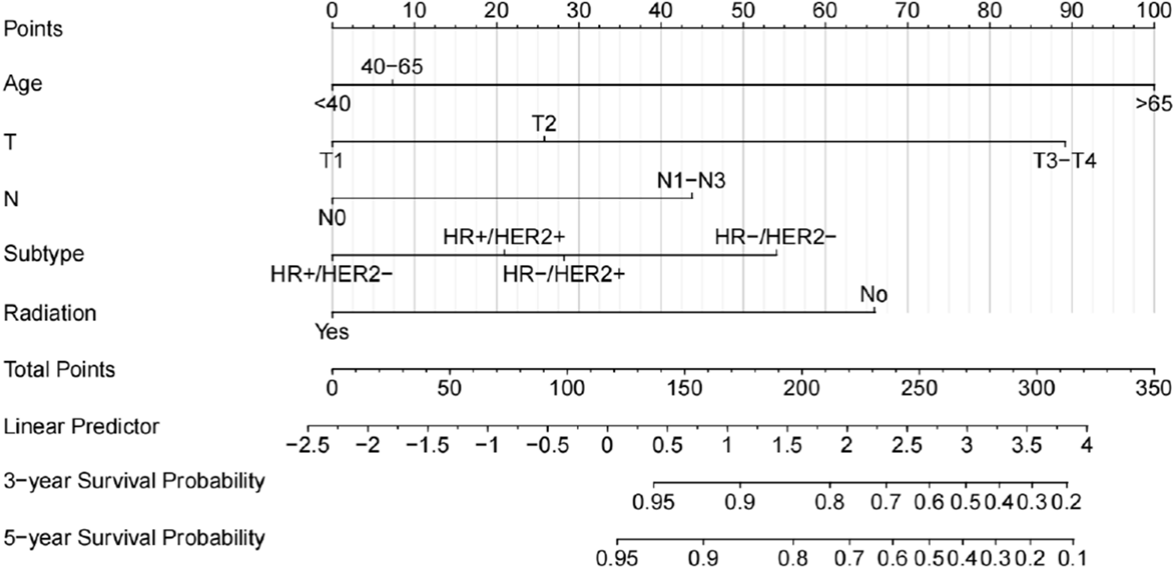
Nomogram for predicting 3- and 5-year OS in patients with MBC. To use the nomogram, an individual patient’s value is located on each variable axis, and a line is drawn upwards to determine the number of points received for each variable value. The sum of these numbers is located on the total points axis, and a line is drawn downwards to the survival axes to determine the likelihood of 3- or 5-year survival.

**Figure 4:**
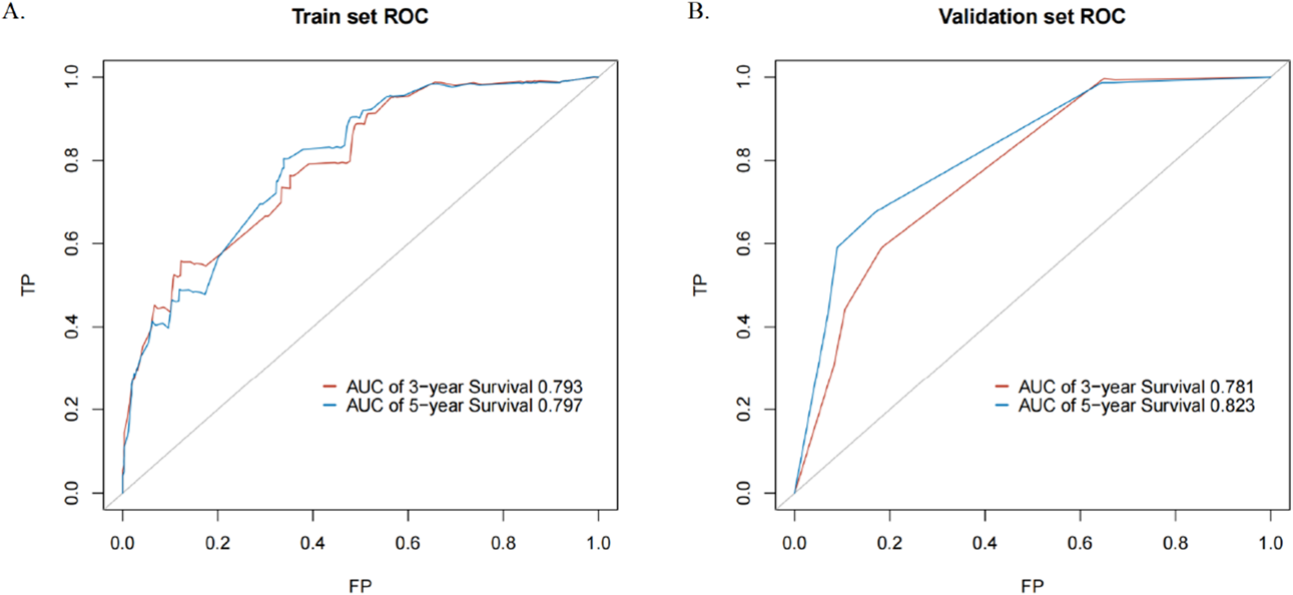
The ROC curves of 3- and 5-year OS of the training set (**A**) and validation set (**B**). *ROC* receiver operating characteristic, *AUC* area under the curve, *TP* true positive rate, *FP* false positive rate.

**Figure 5:**
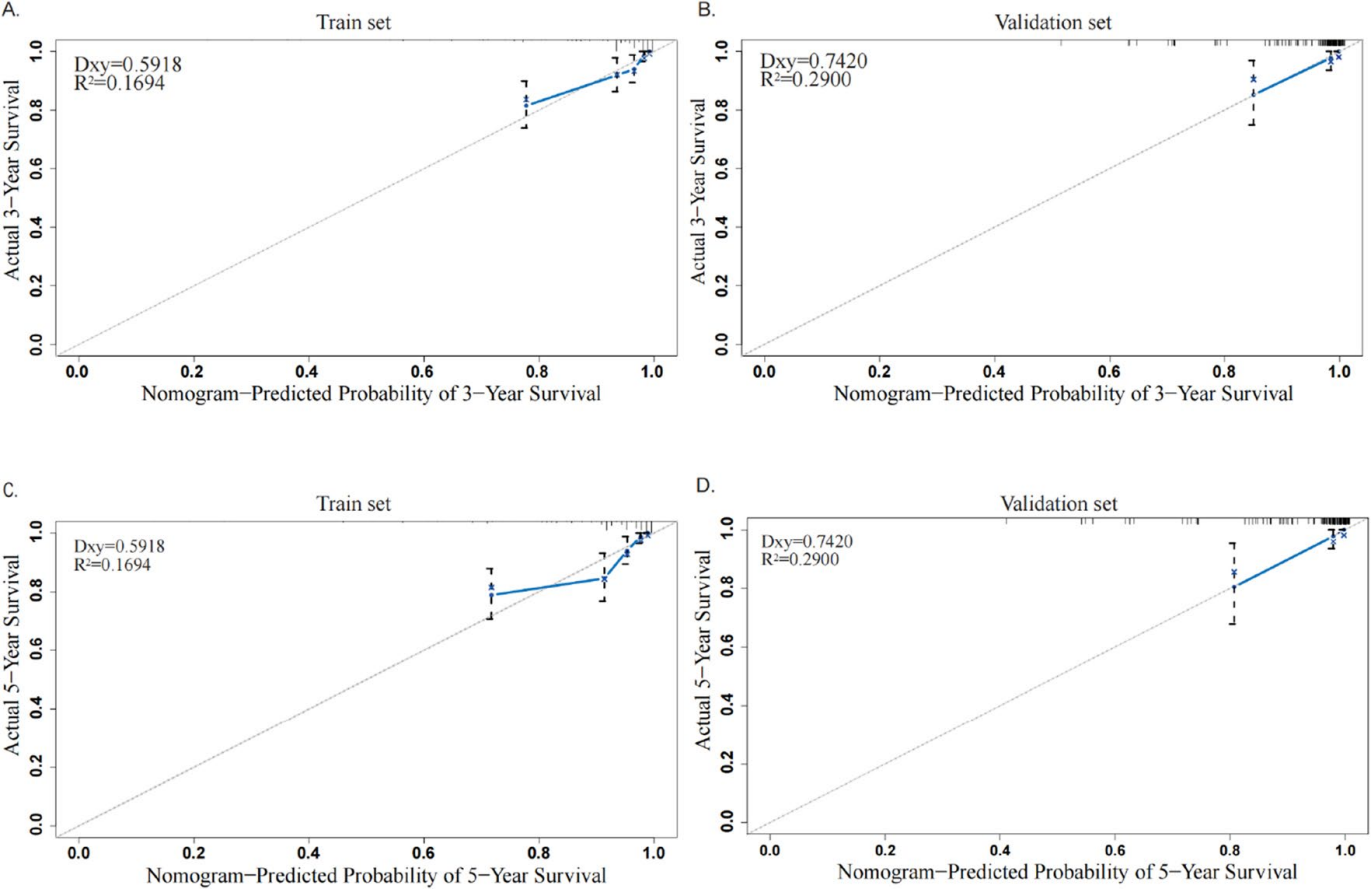
The calibration curve of OS at 3 and 5 years. The calibration curve for predicting patient survival at 3 years (**A**) and 5 years (**C**) in the training set and at 3 years (**B**) and 5 years (**D**) in the validation set. The nomogram-predicted probability of OS is plotted on the x-axis; the actual OS is plotted on the y-axis. The gray dashed lines show the ideal reference line where predicted probabilities would match the observed survival rates. The blue line shows the relationship between actual and predicted survival rate. Blue dots represent apparent calibration accuracy obtained by stratifying into intervals of predicted 0.5-y survival containing 40 events per interval and plotting the mean predicted value within the interval against the stratum’s Kaplan‒Meier estimate. The blue cross represents bootstrap bias-corrected Kaplan‒Meier estimates. Dxy = 2 × (C–0.5). The C-statistic is just AUC. The “R2” index ranges from 0 to 1 and is interpreted as the proportion of variance explained.

We incorporated five independent prognostic factors of OS screened out by the Cox regression model into a nomogram predicting the likelihood of 3- and 5-year OS for the MBC population (Fig. 3). Age at diagnosis was the factor that had the greatest impact on the survival rate, followed closely by T stage, radiation, subtype, and N stage. Next, the nomogram's discriminative power was assessed using the area under the curve (AUC) of receiver operating characteristic (ROC) curves. The AUCs for the 3- and 5-year OS in the training set were 0.793 and 0.797, respectively (Fig. 4A). The AUCs for the 3- and 5-year OS were 0.781 and 0.823, respectively, in the validation set (Fig. 4B). The findings above demonstrated that the nomogram's prediction accuracy was high. At the same time, the calibration plots of the training and validation sets (1000 bootstraps) showed that the nomogram's predicted survival probability was consistent with the actual prognostic outcomes (Fig. 5). To display the application of this nomogram, we included five patients and certain values of the five independent prognostic factors to show the readers how to predict the survival rates at the 3- or 5-year follow-up using the nomogram (Supplemental Table 1).

### Risk stratification analysis Dxy

**Figure 6:**
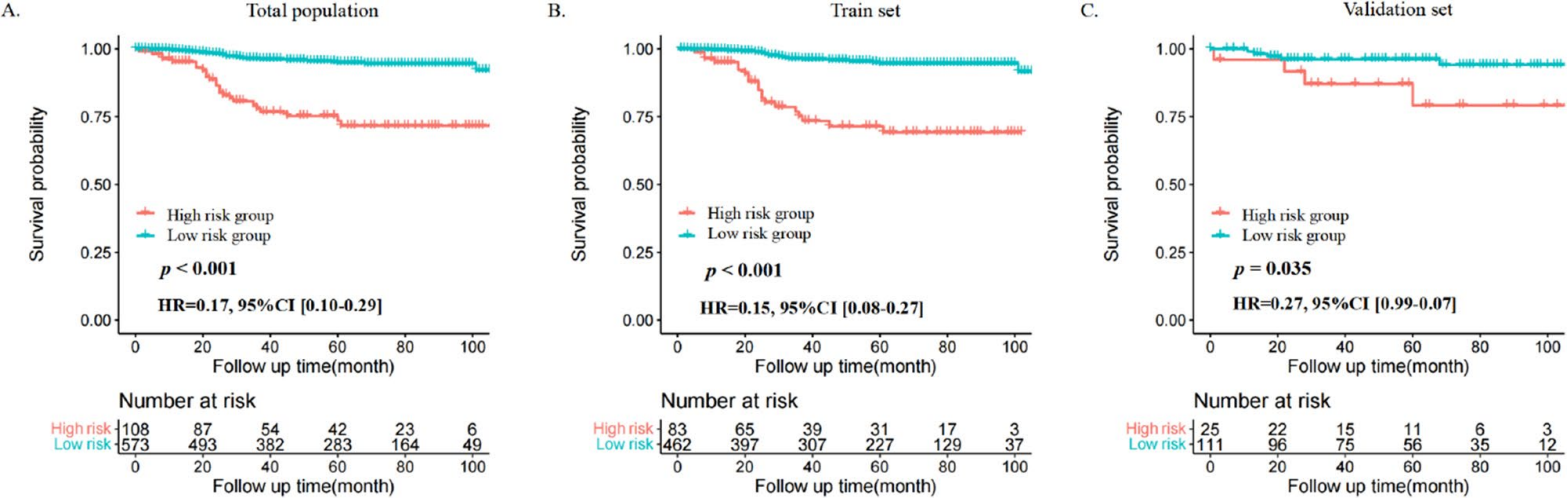
Kaplan‒Meier curves of OS for patients with MBC in the low- and high-risk groups. (**A**) Total population, (**B**) Training set, (**C**) Validation set.

Furthermore, each variable was given a score in accordance with the nomogram (Table 4), and the total score for each patient was obtained. Based on the total nomogram values for each patient, we created a risk classification model. Afterward, the optimal cut-off value of the total score was assessed via X-tile software (Supplemental Fig. 1), and patients with MBC were then divided into a low-risk (573/681, 84.14%, score ≤ 186) group and a high-risk group (108/681, 15.86%, score ≥ 187) based on this optimal cut-off value. Kaplan‒Meier curves were generated in the total population (*p* < 0.001, Fig. 6A), training set (*p* < 0.001, Fig. 6B) and validation set (*p* = 0.035, Fig. 6C), demonstrating that the novel risk stratification framework can accurately distinguish between the two prognostic categories for OS of patients with MBC.

**Table 4:** The risk score of each independent prognostic factor.

Characteristics	Points
Age at diagnosis	
< 40	0
40–65	7
> 65	100
T stage	
T1	0
T2	26
T3–T4	89
N stage	
N0	0
N1–N3	44
Subtype	
TNBC	54
HER2-enriched	28
Luminal A	0
Luminal B	21
Radiation	
No	66
Yes	0

*HER2* human epidermal growth factor receptor 2, *TNBC* triple-negative breast cancer.

### Chemotherapy's effects on the survival benefits in different stratifications

**Figure 7:**
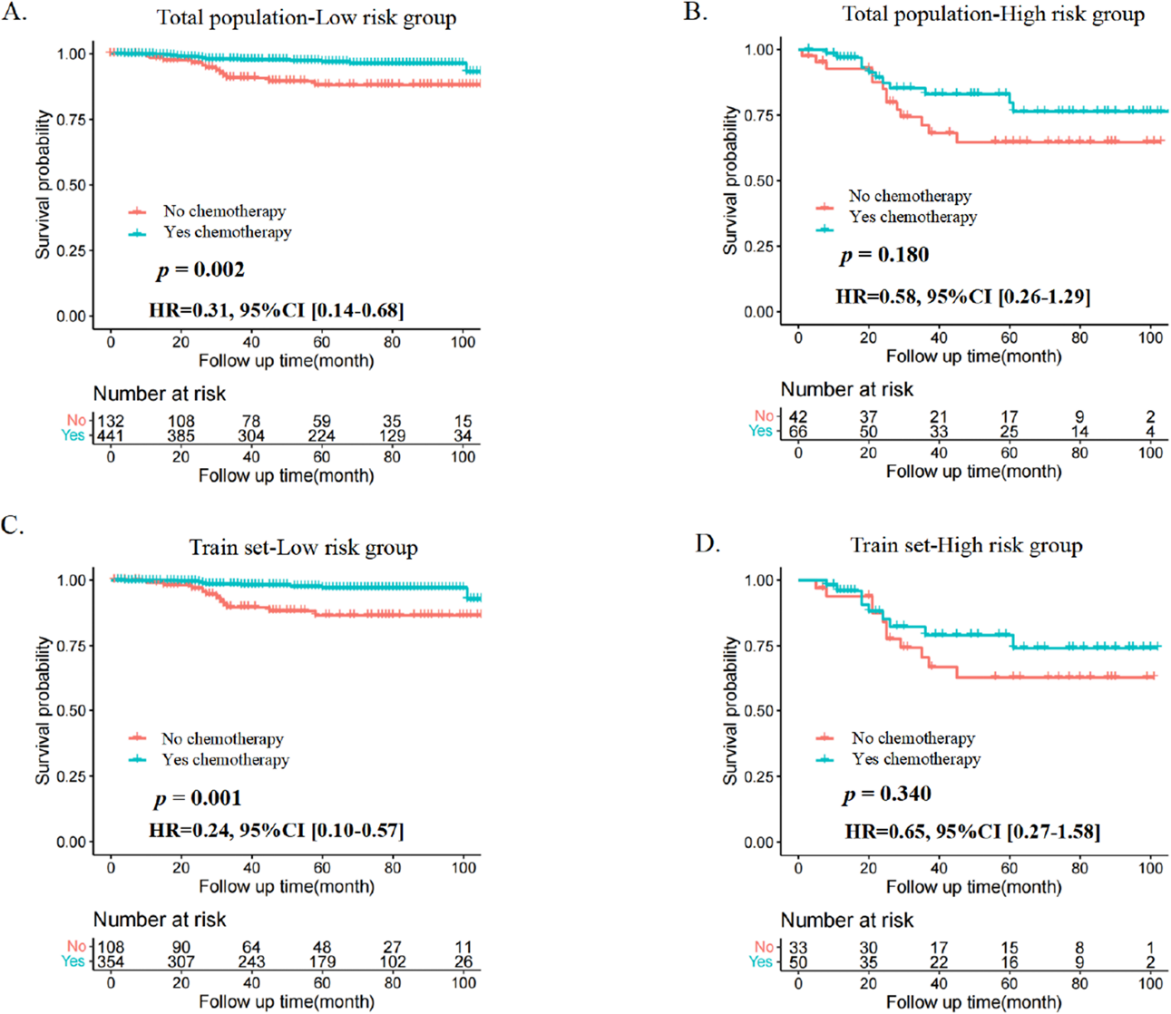
Survival benefits of chemotherapy in low- and high-risk groups of patients with MBC. (**A**,**B**) Total population, (**C**,**D**) Training set.

To further assess the survival benefit of chemotherapy, Kaplan‒Meier curves were generated in the two stratified risk groups. The results showed that in both the total population and the training set, patients with MBC in the low-risk group benefited from chemotherapy (total population: *p* = 0.001, Fig. 7A; training set: *p* = 0.001, Fig. 7C), while no evidence show chemotherapy can improve the OS of patients with MBC in the high-risk group as the result is not statistically significant (total population: *p* = 0.180, Fig. 7B; training set: *p* = 0.340, Fig. 7D).

## Discussion

To the best of our knowledge, our study was the first to construct a new risk-stratified model to screen out patients with MBC who are more likely to obtain an overall survival benefit from chemotherapy. First, five independent prognostic factors were screened out in the training set by the Cox regression model, and their prognostic contribution weights for MBC patients were successively age at diagnosis, T stage, radiation, subtype and N stage. The model's high discriminative performance and stability were shown by the 3- and 5-year OS AUC values (training set: 0.793 and 0.797; validation set: 0.781 and 0.823) and calibration plots. Finally, we divided MBC patients into low- (score ≤ 186) and high- (score ≥ 187) risk groups. The prognosis of patients receiving chemotherapy in the low-risk group was noticeably better than that of patients receiving no chemotherapy; however, a similar chemotherapy benefit was not observed in the high-risk group.

Studies have proven that adjuvant chemotherapy has no effect on the recurrence rate and survival rate of MBC patients^15,16^. In addition, there is evidence that the recurrence and survival of MBC patients are not affected by tumor size or clinical axillary lymph node status^3,16^. Adjuvant chemotherapy significantly improved clinical survival, but only in patients with tumors larger than 2 cm^15^. At this point, it is not accurate to determine whether MBC patients should receive chemotherapy according to TNM stage classification. Nevertheless, the NCCN guidelines recommend that cases diagnosed with MBC be treated as other invasive ductal carcinomas based on tumor size, grade and lymph node status^7^. Therefore, it is necessary to establish a new risk-stratified prediction model for MBC patients to screen out the population benefiting from chemotherapy. The application of such a prediction model in clinical practice can identify patients who benefit from chemotherapy and remind clinicians to adjust the treatment plan in time. However, it is important to emphasize that high risk group has a much smaller sample size than the low-risk, therefore more data should be collected before we can reach a more clear conclusion about if the high-risk will benefit from chemotherapy. For patients who cannot benefit from chemotherapy, systemic treatment with a descending step and local treatment with an ascending step can be carried out.

Additionally, previous studies discovered that patients with MBC had advanced grade, larger tumor size, and a higher proportion of TNBC subtypes but had favorable long-term outcomes^6,8,10,18–25^, which agreed with the findings of our current research. Nodal status and tumor size are still the two most critical prognostic indicators in patients with MBC^10,26^. However, the results of this study suggested that age at diagnosis is a more significant contributor to the new risk prediction model than lymph node status and tumor size. A previous study based on the SEER database also confirmed that age is a prognostic factor for breast cancer-specific death (BCSD) in other IDC patients but not in MBC patients^27^. Another study involving 2001 patients showed that older age was a poor prognostic factor for OS in MBC patients^20^, which was consistent with our results. It is well known that older breast cancer patients have unique physical characteristics, including more comorbidities, shorter life expectancy, and poorer life expectancy than younger breast cancer patients^28,29^. The results of the Early Breast Cancer Trialists' Collaborative Group (EBCTCG) show that the benefit of chemotherapy gradually decreases with age^30^. The adverse effects of chemotherapy and its related mortality increased with age. For example, the incidence and chemotherapy-related mortality of acute myeloid leukemia under 50 years old, 50–64 years old, and over 64 years old were 0.3%, 0.7%, 1.8%, and 0.2%, 0.4%, 1.5%, respectively^31^. Collectively, since elderly breast cancer patients are often associated with medical complications, the risk of death associated with chemotherapy is increased, so the advantages and disadvantages of adjuvant chemotherapy should be weighed in patients with MBC.

Several limitations remain in this study. First, the risk stratification model constructed in this study is based on the SEER database and lacks external validation data. Second, other important variables can affect the outcome of MBC patients, such as a lack of detailed information on the systematic treatments received (endocrine therapy and targeted therapy), specific chemotherapy regimens and toxic side effects in the SEER database. Finally, since only patients with comprehensive clinical features were enrolled in this retrospective cohort study, there may be selection bias in our study. Therefore, additional verification of the applicability of the results to different populations needs to be performed in prospective clinical investigations.

In summary, based on five clinicopathological features (age at diagnosis, T stage, radiation, subtype and N status), this study for the first time constructed a new risk stratification model to screen for chemotherapy survival benefits in MBC patients. These results suggest that clinicians should be more cautious in evaluating chemotherapy for high-risk patients. In the future, larger and more extensive prospective clinical studies will be needed to screen MBC patients for the possibility of exemption from chemotherapy.

## Materials and methods

### Data sources

Data from our study were collected from 18 registries of SEER using the latest SEER*Stat 8.3.8 software, which was built by the National Cancer Institute and updated in November 2018. Since the SEER database is available to global users, informed consent of patients was not required for this study. Therefore, the Ethics Committee of the First Affiliated Hospital of Xi'an Jiaotong University is exempted from review.

### Patient and variable selection

Inclusion criteria: (1) female patients; (2) pathologically diagnosed as MBC (ICD-0-3 8510/3); (3) diagnosed between 2010 and 2018. The reason we enrolled patients with MBC diagnosed in the SEER database during 2010–2018 is that HER2 status information was recorded after 2010.

Exclusion criteria: (1) patients with unknown race, marital status or grade; (2) patients with unknown T stage or T0 or Tis, unknown N status (derived AJCC 7th ed (2010–2015) and Derived SEER Cmb Stg Grp (2016+); (3) patients with unknown M status or M1; and (4) patients with unknown/borderline status of estrogen receptor (ER), progesterone receptor (PR), HER2. Positive staining of more than or equal to 1% of tumor cells was defined as ER/PR positivity. HER2 status was confirmed by immunohistochemistry (IHC) or fluorescence in situ hybridization (FISH) assays. HER2 IHC scores of 0 and 1+ were considered HER2 negative, and a HER2 score of 3+ was considered HER2 positive, whereas a HER2 score of 2+ was further estimated by FISH, and HER2 gene amplification was considered HER2 positive.

A screening flow diagram of our study population is presented in Fig. 1. A total of 507 patients receiving chemotherapy and 174 patients not receiving chemotherapy who met the inclusion criteria were enrolled in our study.

### Outcomes

OS was determined as the interval from diagnosis to death from any cause and served as this study's primary endpoint. The second endpoint of the study was BCSS, which was determined as the interval between initial diagnosis and death from breast cancer.

### Statistical analysis

Fisher’s exact test or Pearson’s chi-square test was utilized to compare the differences in clinicopathological features between groups. PSM^30^ was executed to eliminate differences in age at diagnosis, marital status, T stage, N status and radiation between groups at a 1:1 ratio, and the calliper width of PSM was set as 0.05. All eligible patients were randomly split into training or validation sets at a ratio of 8:2 before PSM.

We used univariate and multivariate Cox proportional hazard models to distinguish independent prognostic elements in the training set and evaluated the related hazard ratios (HRs) and 95% confidence intervals (CIs) for each prospective risk factor. Then, a nomogram was established to predict 3- and 5-year OS based on the results of the multivariate Cox regression model utilizing the “rms” and “survival” R packages. The nomogram was constructed by proportionally scaling each regression coefficient from 0 to 100 in multivariate Cox regression. One hundred points are given to the influence of the variable with the highest coefficient β (absolute value). The total points are calculated by adding the points for all independent variables. These total points are then transformed to predicted probabilities. The AUCs of the ROC curves and calibration plots (1000 bootstrap resamples) were used to evaluate the nomogram's discriminative power and predictive accuracy, respectively. Afterward, based on each patient's overall score from the nomogram, a risk categorization method was developed. Next, all patients were separated into low- and high-risk groups according to the best cut-off value for each patient's total score, which was determined using X-Tile software^31^. Finally, Kaplan–Meier curves and log-rank tests were conducted to analyse the OS or BCSS of patients between the groups.

All statistical analyses were performed in R studio (v4.1.1) and SPSS 25 (SPSS, Chicago, IL), and statistical significance was set at a p value of < 0.05.

### Ethical approval

This is an observational study. The First Affiliated Hospital of Xi'an Jiaotong University Research Ethics Committee has confirmed that no ethical approval is required.

## Supplementary Information

Supplementary Information.

## Data Availability

The datasets presented in this study can be found in online repositories. The names of the repository/repositories and accession number(s) can be found below: https://seer.cancer.gov.
