# Supplementary material for: A risk stratification model to predict chemotherapy benefit in medullary carcinoma of the breast: a population-based SEER database
Source: Sci Rep. 2023 Jul 3;13:10704. doi: 10.1038/s41598-023-37915-2 (PMC10317966; doi:10.1038/s41598-023-37915-2)
Supplement: Supplementary file 1 — Supplementary Information. [file 41598_2023_37915_MOESM1_ESM.doc]

**Supplementary material**

Supplemental table 1:

Supplemental table 1. The application of the risk stratification nomogram.

| Characteristics | Points | Patients | | | | |
| --- | --- | --- | --- | --- | --- | --- |
| Age at diagnosis |  | 1 | 2 | 3 | 4 | 5 |
| <40 | 0 |  | √ |  |  |  |
| 40-65 | 7 | √ |  |  |  |  |
| >65 | 100 |  |  | √ | √ | √ |
| T stage |  |  |  |  |  |  |
| T1 | 0 | √ | √ |  |  |  |
| T2 | 26 |  |  |  |  |  |
| T3-T4 | 89 |  |  | √ | √ | √ |
| N stage |  |  |  |  |  |  |
| N0 | 0 |  | √ |  |  |  |
| N1-N3 | 44 | √ |  | √ | √ | √ |
| Subtype |  |  |  |  |  |  |
| TNBC | 54 | √ |  | √ |  |  |
| HER2-enriched | 28 |  |  |  | √ |  |
| Luminal A | 0 |  |  |  |  | √ |
| Luminal B | 21 |  | √ |  |  |  |
| Radiation |  |  |  |  |  |  |
| No | 66 | √ |  |  |  | √ |
| Yes | 0 |  | √ | √ | √ |  |
| Total point | | 171 | 21 | 287 | 261 | 299 |
| 3-year survival probability | | 91% | 99% | 39% | 56% | 30% |
| 5-year survival probability | | 87% | 99% | 28% | 45% | 19% |
| Risk group | | low risk | low risk | high risk | high risk | high risk |

**Note:** HER2, human epidermal growth factor receptor 2; TNBC, triple-negative breast cancer.

**Supplemental figure 1:**


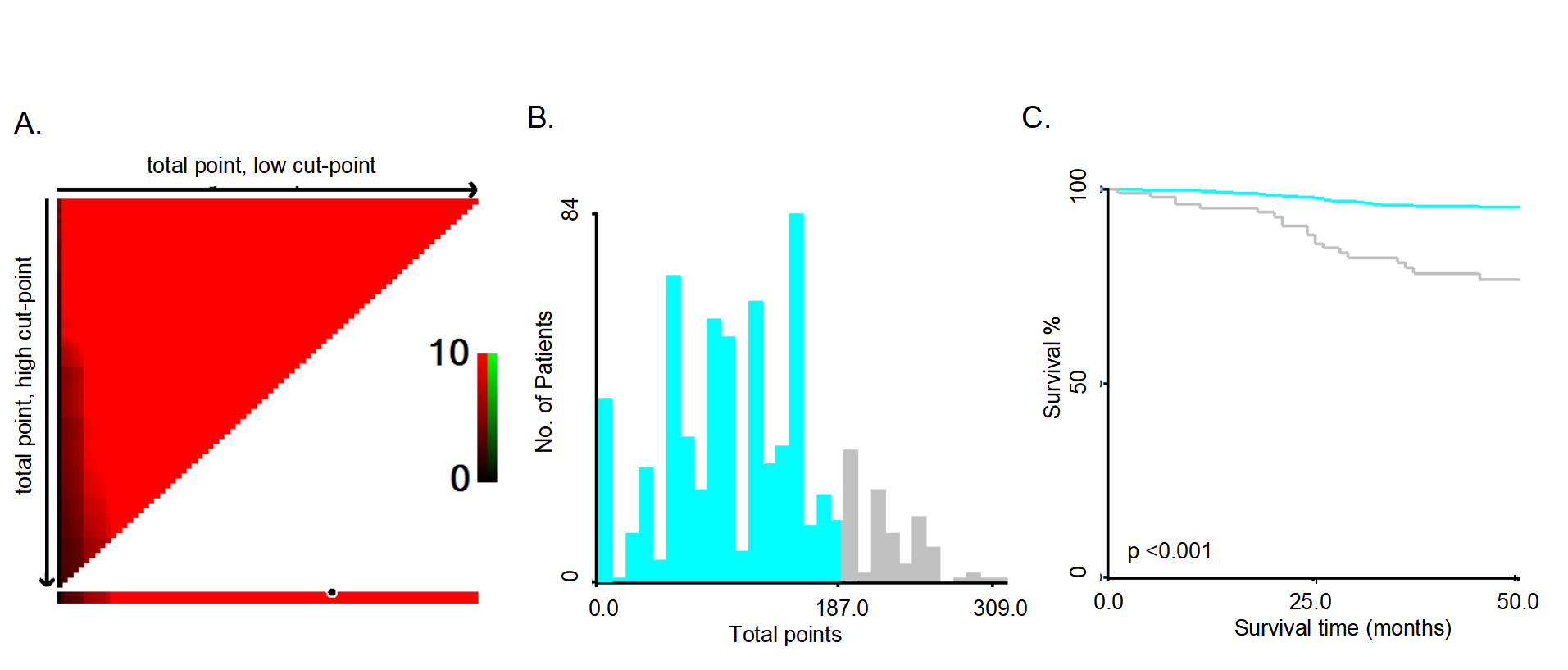


**Supplemental figure 1. The optimal cut-point by the X-tile.** The plot shows the χ^2^ log-rank values produced when dividing the cohort with one cut-point, producing high and low subsets. The X-axis represents all potential cut-points from low to high (left to right) that define a low subset, whereas the Y-axis represents cut-points from high to low (top to bottom) that define a high subset (A). The arrows represent the direction in which the low subset (X-axis) and the high subset (Y-axis) increase in size. Red colouration of cut-points indicates an inverse correlation with survival, whereas green colouration represents direct associations. The optimal cut-point occurs at the brightest pixel (green or red). The cut-point highlighted by the black/white circle in the left panels is shown on a histogram of the entire cohort (middle panels) and a Kaplan‒Meier plot (right panels; low risk subset = blue, high risk subset = gray).
